# The dynamic regenerative scaffold stenting and shielding hernia system for dissection-free, atraumatic hernioplasty. Results of an experimental animal study

**DOI:** 10.1038/s41598-025-24487-6

**Published:** 2025-10-31

**Authors:** Giuseppe Amato, Giorgio Romano, Luca Cicero, Giovanni Cassata, Giuseppe Di Buono, Giorgio Romano, Vito Rodolico, Roberto Puleio, Antonino Agrusa

**Affiliations:** 1 Department of Precision Medicine in Medical, Surgical and Critical Care (Me.Pre.C.C.), University of Palermo, Via del Vespro, 129, Palermo, 90127 PA Italy; 2 CEMERIT-Experimental Zooprophylactic Institute of Sicily, Palermo, Italy; 3 Postgraduate School of General Surgery, University of Palermo, Palermo, Italy; 4 Department PROMISE, Section Pathological Anatomy, University of Palermo, Palermo, Italy; 5 Department of Pathologic Anatomy and Histology, IZSS Palermo, Palermo, Italy

**Keywords:** Abdominal hernioplasty, Dissection free, Hernia prostheses, Laparoscopic hernia repair, Regenerative scaffolds, Porcine experimental model, Preclinical research, Medical research, Biotechnology, Biomedical materials, Implants

## Abstract

Conventional hernia repair using static flat meshes is often associated with complications casused by extensive dissection, implant fixation, and poor biological response. The Stenting & Shielding (S&S) Hernia System offers a novel solution designed for dissection-free, fixation-free hernia repair. The S&S Hernia System has been evaluated in a porcine model, focusing on procedural simplicity, device retention and regenerative properties. The experimental study involved 10 pigs, each implanted with two S&S devices. Follow-up assessments included ultrasound, laparoscopic examinations, and histological analyses over a period ranging from 4 weeks to 8 months post-implantation. The S&S Hernia System, made from medical-grade polypropylene-based thermoplastic elastomer (TPE), features a rayed 3D scaffold and an oval shield connected by a mast. Delivered and positioned intraabdominally without dissection, the device transforms into a self-retaining scaffold to obliterate the hernia defect. Primary outcomes included ease of delivery, visceral adhesion formation, and device retention, ensuring no dislocation. Secondary outcomes evaluated histological evidence of tissue regeneration, including the development of connective tissue, muscles, vessels, and nerves within the scaffold. Immunohistochemistry was used to detect tissue growth factors promoting regeneration of the abdominal wall’s typical components. All 20 implanted devices remained securely in place without dislocation. Transient adhesion bands were observed in two shields at 1 month but resolved by 3 months. No visceral adhesions were detected at the time of animal sacrifice. Histological analyses demonstrated tissue regeneration within the scaffold, while immunohistochemistry confirmed the presence of growth factors supporting regeneration. Overall, the S&S Hernia System showed promising results in a porcine model, with no dislocation and evident regenerative potential. Its rapid, dissection-free delivery and compliance with abdominal wall dynamics simplify the procedure while minimizing adhesions. These findings warrant further clinical investigation to validate its application in human patients.

## Introduction

Abdominal wall hernias are a common condition associated with significant morbidity and complications. Despite numerous surgical techniques, there is no consensus on a gold standard for hernia repair. Many current treatment strategies fail to address the underlying pathophysiology and disrupt the natural dynamics of the abdominal wall.

Conventional hernia repair primarily relies on flat meshes designed to “reinforce” the abdominal wal. However, these static and passive meshes present several limitations:

1. They do not replicate the dynamic function of abdominal musculature, requiring fixation that exacerbates postoperative pain, impairs movement, and results in unphysiological outcomes^1–4^.
2. Conventional flat meshes primarily elicit a fibrotic foreign body reaction^5–9^, which in some cases may also include granulomatous features, and nevertheless fail to address the degenerative origins of hernia disease, such as the chronic compressive damage to the abdominal wall musculature commonly observed in inguinal hernias^10–14^.
3. The hernia defect often remains patent post-repair, increasing the risk of recurrence.
4. Placement typically necessitates extensive tissue dissection, raising the risk of iatrogenic injuries, edema, and delayed recovery.

An optimal hernia repair approach should avoid fixation to respect abdominal wall physiology, promote tissue regeneration to restore the muscular barrier, achieve permanent defect obliteration to prevent recurrence, and simplify the procedure to eliminate extensive tissue dissection and surgical trauma during the procedure.

To address these requirements, our group has pioneered the development of the first dynamic regenerative scaffold for inguinal hernia repair^15^. This first-generation device, ProFlor, demonstrated that a three-dimensional, dynamically compliant scaffold could induce tissue regeneration and provide durable defect closure without the need for fixation. However, its use was limited to anterior open implantation or preperitoneal placement via a laparoscopic TAPP procedure^16,17^.

Building upon this foundation, the Stenting & Shielding (S&S) Hernia System was conceived as the second-generation evolution of this concept. While maintaining the regenerative and dynamic compliance principles of ProFlor, the S&S device introduces a novel configuration that allows exclusive laparoscopic intra-abdominal placement eliminating the need of peritoneal and sac dissection, combining defect stenting with a shielding component to protect against intra-abdominal forces. This advancement shortens and simplifies the surgical procedure, broadens the potential applicability across hernia types, and enhances atraumatic implantation.

Summarizing, the S&S Hernia System:

- Provides fixation-free deployment without tissue dissection;
- Permanently obliterates the hernia defect;
- Assures dynamic compliance with abdominal wall movements;
- Acts as a regenerative scaffold, promoting the development of newly formed connective tissue, muscles, vessels, and nerves to restore the integrity of the abdominal wall barrier.

An additional requirement for this new concept was the development of a surgical technique with the shortest possible learning curve, making the approach feasible in all clinical settings.

The device, fabricated from medical-grade polypropylene-based thermoplastic elastomer (TPE), was inspired by cardiovascular stents. Upon deployment, its stent-like component transforms into a dynamic 3D scaffold that adapts to abdominal wall movements fostering a probiotic biological response. An oval shield stabilizes the scaffold and protects against future herniation near the primary defect.

This study presents the results of an experimental trial of the S&S Hernia System in a porcine model, where 20 devices were implanted in 10 pigs and evaluated at defined postoperative intervals.

## Materials and methods

### Ethical approval and study design

The experimental trial was conducted in compliance with the Animal Care Protocol for Experimental Surgery, authorized by the Italian Ministry of Health (Decree No. 379/2021-PR, June 1, 2021). The work has been reported in accordance with the ARRIVE guidelines (Animals in Research: Reporting In Vivo Experiments)^18^.

From February 2022 to November 2024, 10 female pigs (4–6 months old, 40–60 kg) underwent laparoscopic intraabdominal implantation of two Stenting & Shielding (S&S) Hernia Devices. Procedures were performed under general anesthesia (zolazepam + tiletamine 6.3 mg/kg + xylazine 2.3 mg/kg; induction: propofol 0.5 mg/kg; maintenance: isoflurane + pancuronium 0.07 mg/kg). Postoperative care included oxytetracycline (20 mg/kg/day for 3 days).

### Stenting and shielding hernia system: design and function

The S&S Hernia System, composed of medical-grade polypropylene-based thermoplastic elastomer (TPE), whose technical properties are highlighted in Table 1.

**Table 1:** Mechanical properties of the tpe material used for the injection molding of the stenting and shielding hernia system.

TPE mechanical properties	Value	Unit	Test standard
ISO data			
Tensile strength	16	MPa	ISO 37
Strain at break	650	%	ISO 37
Compression set at 70 °C, 24 h	54	%	ISO 815
Compression set at 100 °C, 24 h	69	%	ISO 815
Tear strength	46	kN/m	ISO 34-1
Shore A hardness	89	–	ISO 7619-1
Density	890	kg/m^3^	ISO 1183

The S&S device was designed for permanent defect obliteration. Its components included:

- **Mast element**: Equipped with a button-like distal end and two conic stops.
- **Rayed structure**: Eight rays, each measuring 4.5 cm, connected by a central ring.
- **Assembly ring**: Secured the mast and rayed structure.
- **3D oval shield plate**: Dimensions: 10 × 8 cm; central ring for attachment.

**Fig. 1:**
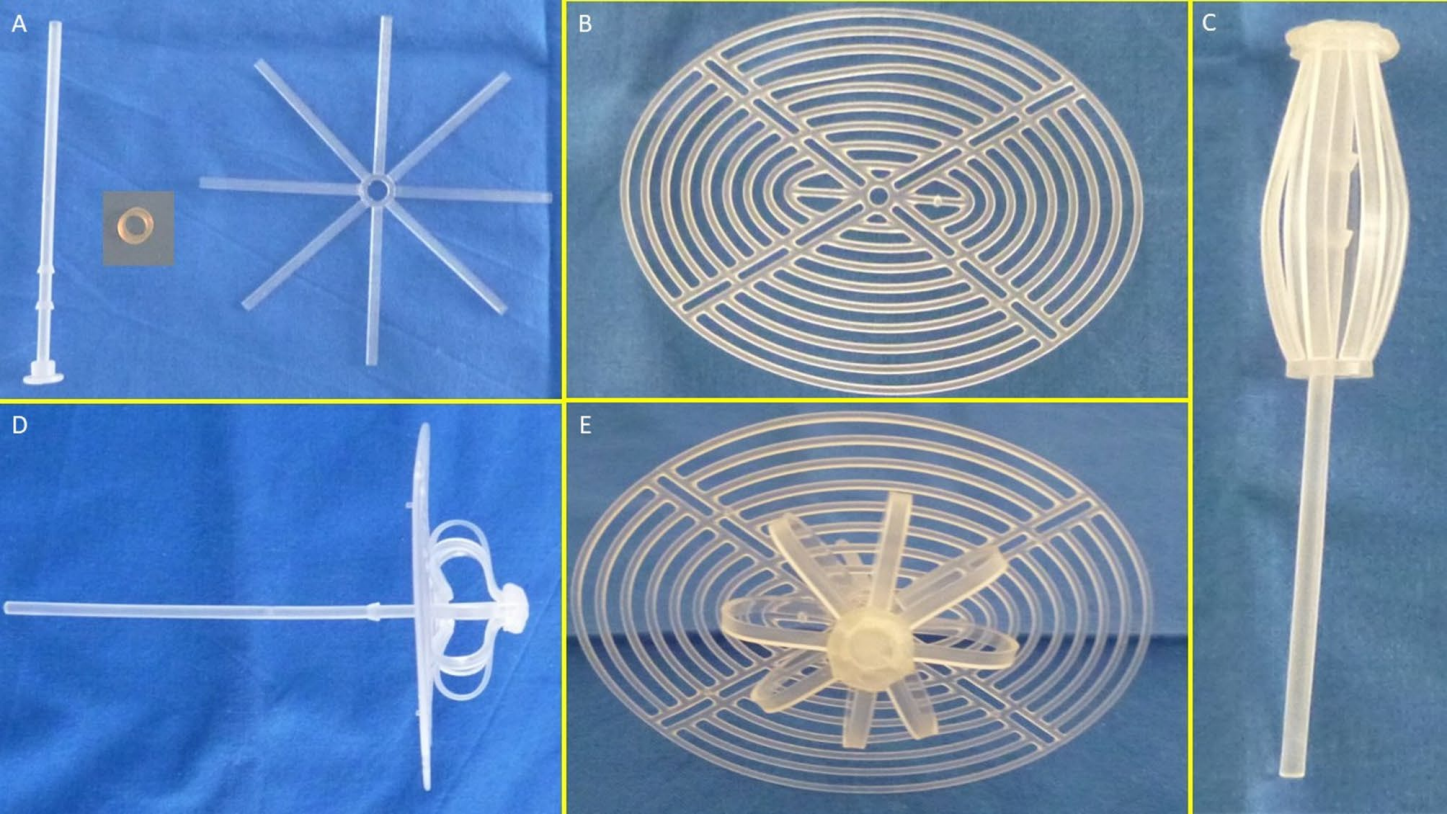
(**A**) The three core elements of the S&S device: The mast element with a button-like enlargement at the distal end and two conic enlargements (stops) just before the distal end. The eight-rayed structure with a central connecting ring. The separate ring intended to firmly assemble the rayed structure with the mast. (**B**) The 3D oval shield plate with a central ring. (**C**) The three components assembled together around the central mast to form a cylindrical compound. (**D**) Sagittal view of the S&S Hernia System in its deployed configuration, showing the shield carried beyond the second conic enlargement (stop) of the mast. (**E**) Frontal view of the S&S Hernia System in its deployed configuration.

Figures 1A and B show the components of the device.

### Operational steps

**Fig. 2:**
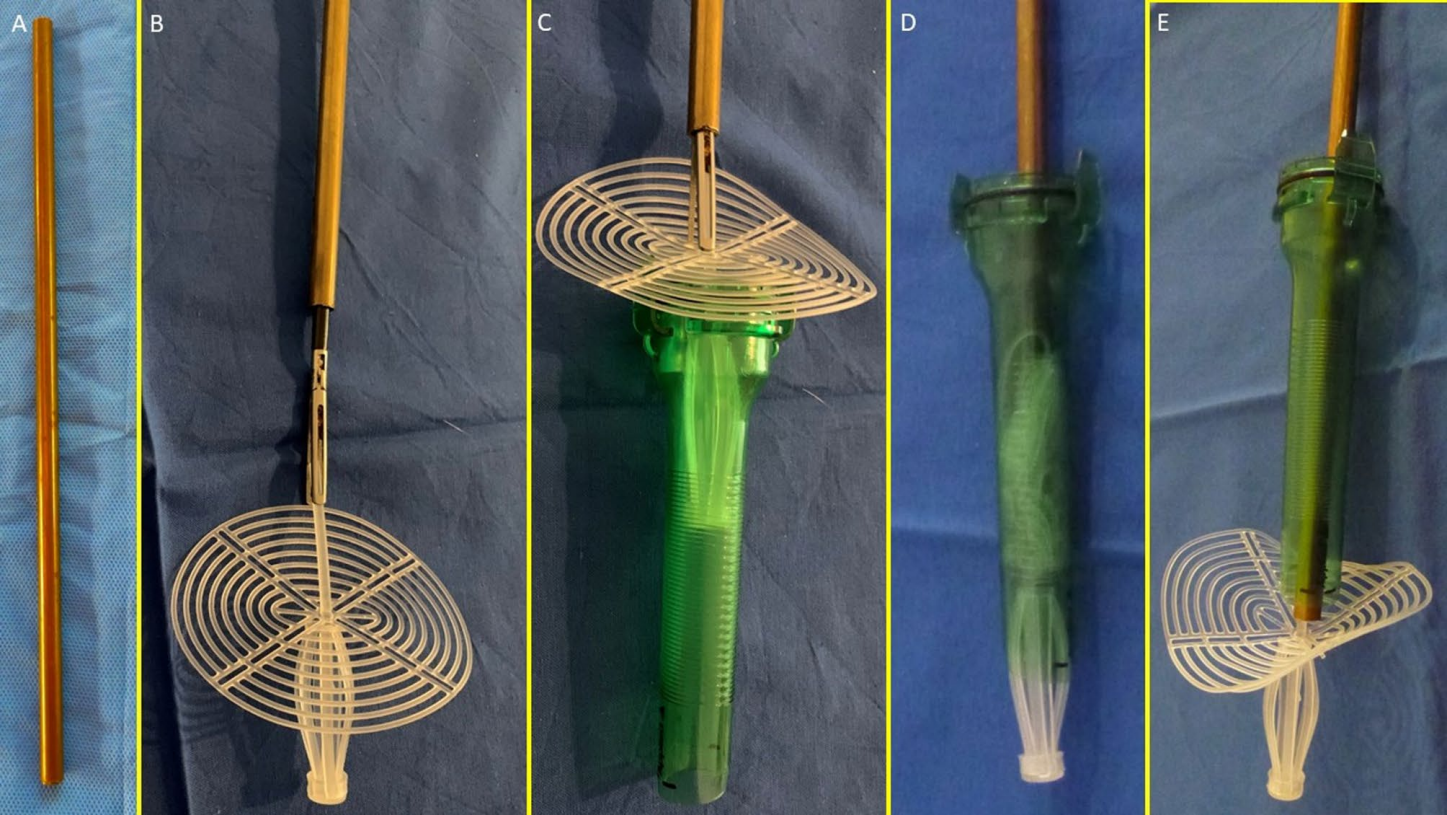
The following figures highlight the delivery model of the Stenting & Shielding Hernia System. (**A**) The metallic tube used to deliver the S&S Hernia System through the trocar channel. (**B**) The S&S device in delivery configuration held by forceps already inserted in the metallic tube. (**C**) The cylindrical part of the S&S device already inside the trocar channel, with the shield about to be introduced for delivery into the abdominal cavity. (**D**) All components of the S&S device are inserted into the trocar while the metallic tube pushes the compound through the channel. (**E**) All components of the S&S device are funneled through the trocar channel. Note the transient deformation of the shield, which is destined to quickly regain its original shape.

**Fig. 3:**
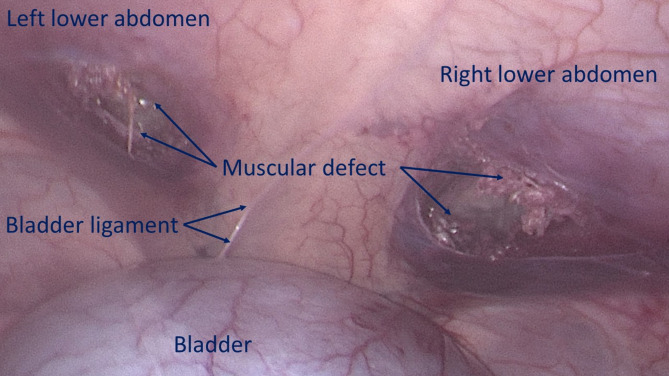
Bilateral defects created in the lower aspect of the abdominal wall, mimicking two hernia openings.

**Fig. 4:**
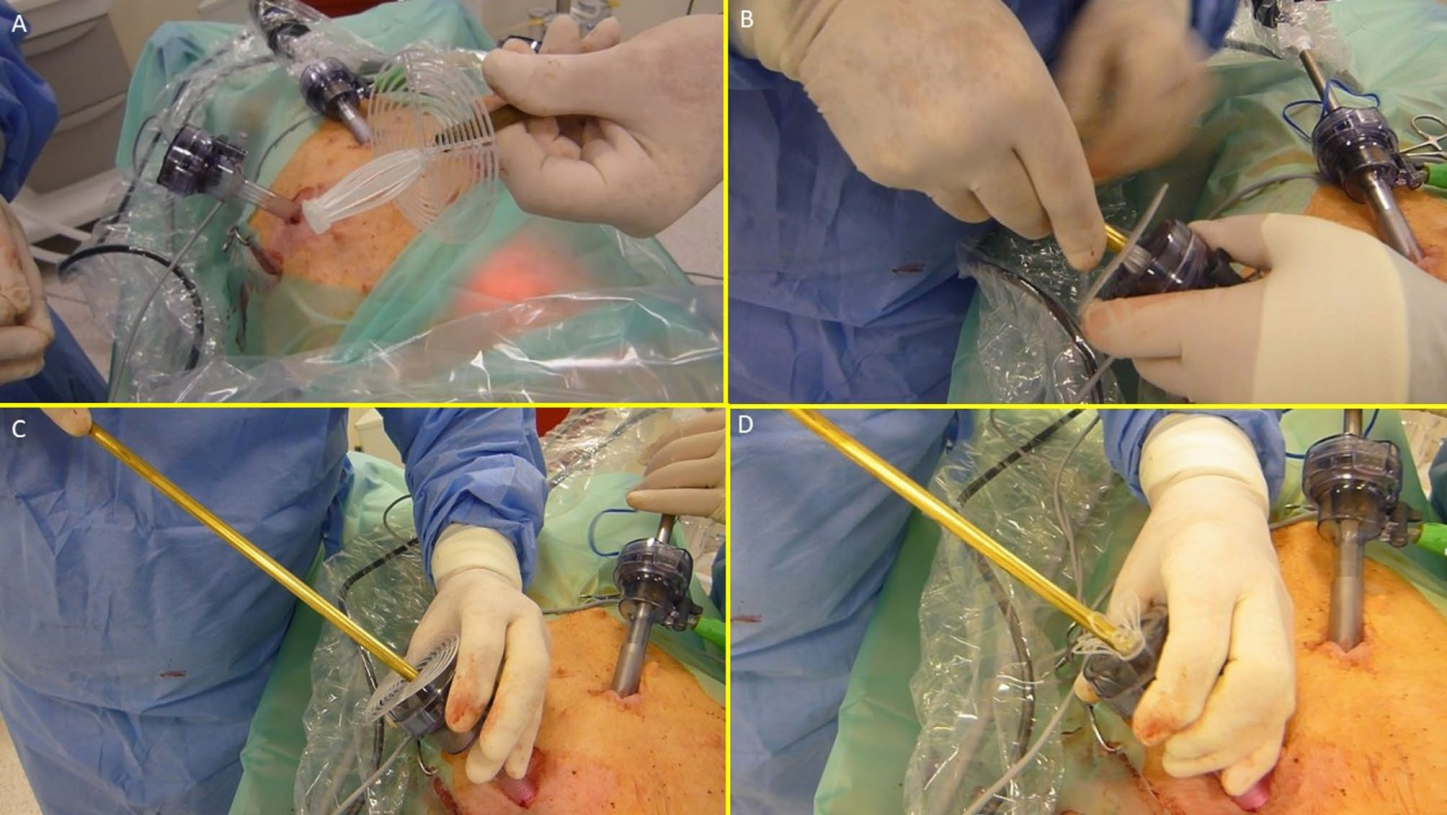
The image sequence (**A**–**D**) shows the external steps of the delivery procedure of the S&S device.

**Fig. 5:**
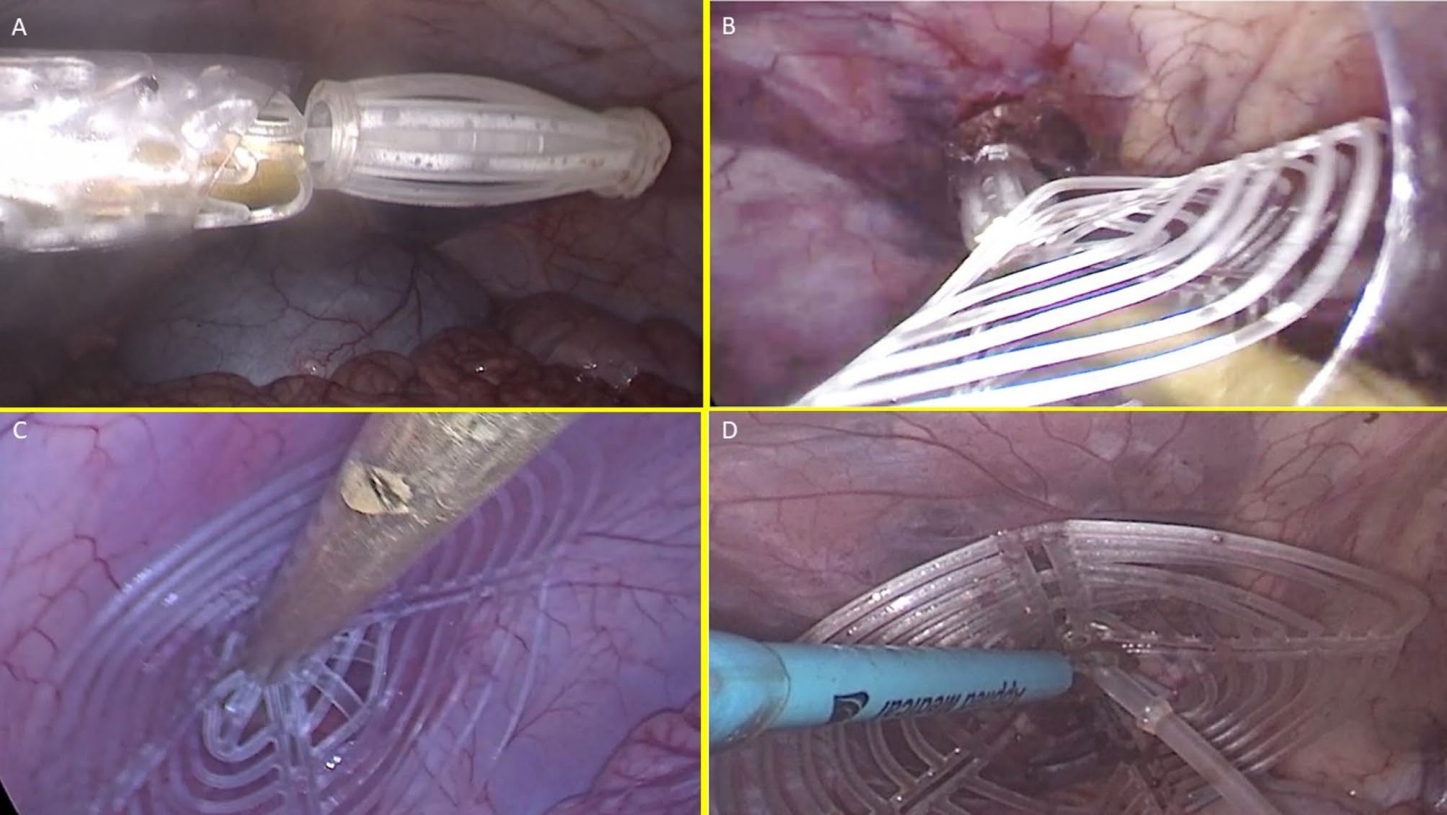
The image sequence (**A–D**) highlights the intraabdominal steps of the delivery procedure of the S&S device.

1. Assembly and preparation:

- The mast, rayed structure, and assembly ring were assembled into a cylindrical compound. (Fig. 1C)
- The shield plate was loosely attached to the mast for laparoscopic delivery.
- The conic enlargements of the mast that served as stops, secured the shield and the 3D scaffold in its final position preventing the shield from slipping backwards. (Fig. 1D – E) ,
- The prepared device, held by the forceps inserted in the metallic tube, was intended to be introduced into a trocar channel to be delivered into the abdominal cavity. (Figs. 2A – E)

2. Deployment:

- All procedures were planned to be performed laparoscopically.
- The first step involved creating a defect in the lower abdominal wall musculature of the pig. Depending on the anatomical aspect of the swine, the defects were made using electrocautery bilaterally either in the lower part of the rectus muscle or in the lower medial aspect of the lateral abdominal musculature to achieve a ca. 3,5 cm wide opening. (Fig. 3)

- The device was compounded by inserting the grip of a laparoscopic forceps into an 8 mm metallic tube. Additionally, the branches of the forceps firmly held the mast of the 3D scaffold in a restrained mode along with the loose shield. (Fig. 4A)

- With this configuration, the S&S device was delivered through a 12 mm trocar into the abdominal cavity (Figs. 4B, C and D and 5A),

- At this stage the device was approached and introduced into the defect. (Fig. 5B)
- Then, with a combined maneuver, the metallic tube was pushed to slide the shield forward along the central mast while simultaneously the forceps pulled the proximal edge of the mast backward. This allowed the shield to surpass the two conic stops of the mast.
- After deployment the 3D scaffold of the device measured 4.5 cm in diameter,

3. Securing the device:

- The conic stops locked the shield in position. Adjustments allowed tailored scaffold expansion at discretion of the surgeons. (Fig. 5C)
- It should be emphasized that the primary retention mechanism of the S&S Hernia System is provided by the interaction between the conic stops of the mast and the shield, which surpassed the stop and locked itself securely in place (Fig. 1D). The subsequent outline of the device in its deployed mode (Fig. 1E) demonstrated a stable configuration. The expansion of the stent within the hernia channel represented only a secondary stabilizing factor, complementing the stop/shield locking system, which was the true safeguard against migration or dislodgement. The redundant mast was excised after confirming stability (Fig. 5D).

**Fig. 6:**
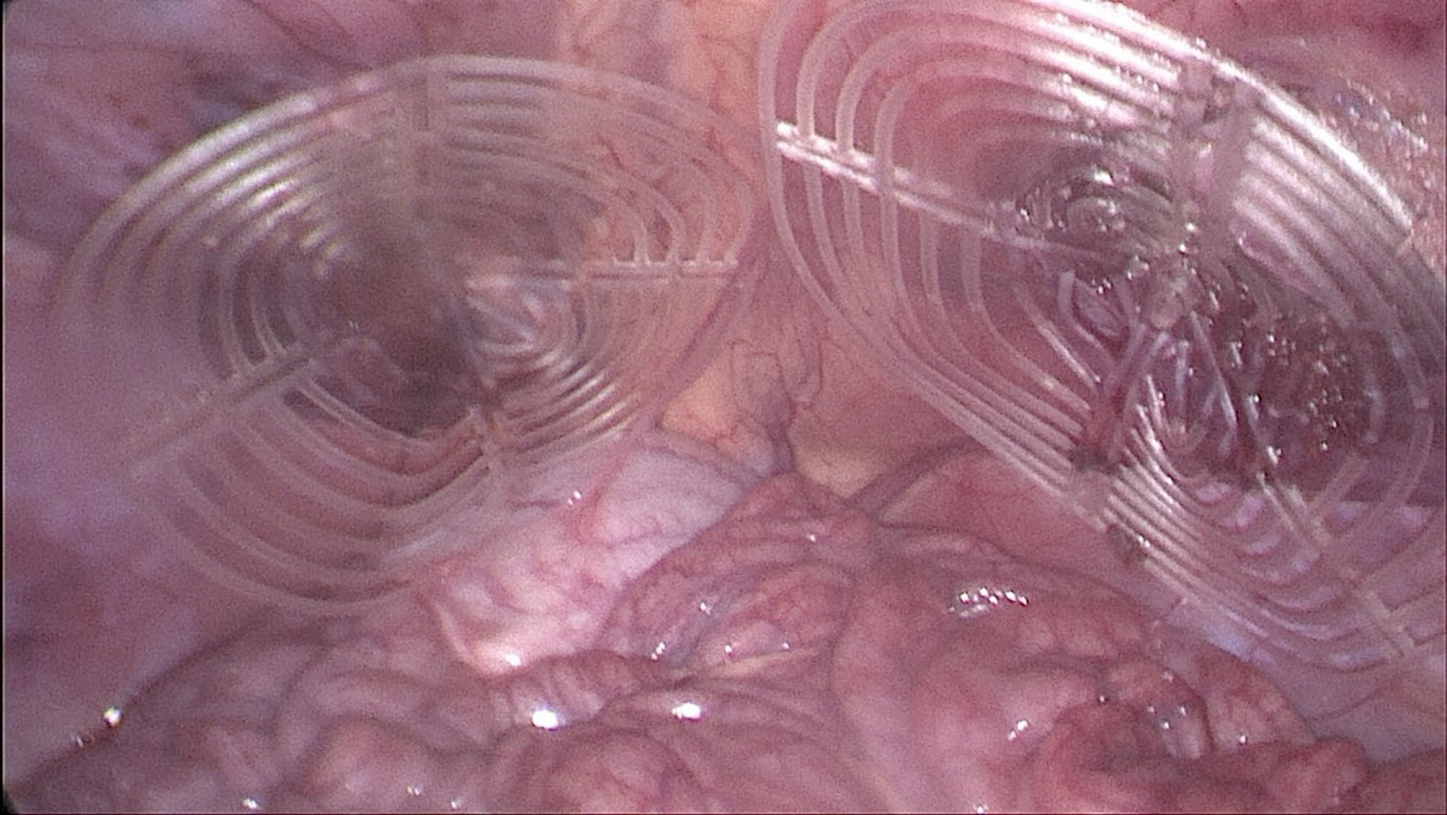
After placement of the 3D scaffold into the defects, the shields of the S&S device cover the surface of the lower abdominal wall bilaterally.

Two S&S devices were implanted in each pig, with final configurations shown in Fig. 6.

### Follow-up protocol

Euthanasia and follow-up evaluations were planned as follows:

- **Short-term** (1 month): 2 pigs.
- **Midterm** (3–4 months): 3 pigs.
- **Long-term** (5–8 months): 5 pigs.

Controls included ultrasound and laparoscopic evaluations of the device for positional stability, shield integration, and absence of adhesions to abdominal viscera. Euthanized animals underwent laparotomy for macroscopic assessment and en bloc removal of the S&S device for histological and immunohistochemical analysis.

### Histological and immunohistochemical study

Excised devices were fixed in 10% neutral buffered formalin and sectioned for histological analysis using Hematoxylin-Eosin (H&E) and Azan-Mallory trichrome staining (Bio-Optica Milano, Italy). Key features examined included inflammatory response, vascularization, and documentation of the ingrown tissue.

Immunohistochemistry assessed growth factors and specialized tissue development:

1. **Neo-neurogenesis**: NSE (Neuron-specific Enolase, 1:100, LSBio, USA).
2. **Neo-angiogenesis**: VEGF (1:50, R&D Systems, USA); PECAM-1 (CD31, 1:50, Dako-Agilent, USA).
3. **Vascular maturity**: SMA (Smooth Muscle Actin, 1:100, Dako-Agilent, USA).
4. **Neomyogenesis**: NGF (1:100, Abcam, USA).
5. **Neurogenesis markers**: NGFRp75 (1:100, Santa Cruz Biotechnology, USA).

### Endpoints

The study endpoints were classified into two arms:

1. Surgical Outcomes:

- Feasibility of laparoscopic delivery without tissue dissection.
- Time required for deployment.
- Learning curve for optimal procedure time.
- Device retention within the defect.
- Ultrasound-documented tissue incorporation.
- Laparoscopic evidence of presence/absence of visceral adhesions.

2. Biological Response:

- Macroscopic ingrowth within the 3D scaffold.
- Histological evaluation of tissue integration and inflammatory response.
- Immunohistochemical detection of growth factors (e.g., VEGF, NGF) supporting vascular, muscular, and neural regeneration.

## Results

### Outcomes of the surgical procedure

All procedures were performed without intraoperative complications. Following the creation of bilateral muscular defects in the lower abdomen, no further dissection or additional tissue trauma inside the abdominal cavity was necessary. The S&S devices were delivered through a 12 mm trocar using the proprietary tool that safely guided the device to the defect site. The combined maneuver to position and expand the device within the defect proved to be simple and uncomplicated, allowing the 3D scaffold to be securely fixed according to the procedural plan.

Initially, the delivery and placement process took 3–6 min, from trocar introduction to final adjustment. However, starting with the 7th implantation attempt, the time decreased significantly, averaging 1 min per deployment.

### Surgical follow-up

All animals, except one, survived until their planned explantation dates. One pig, which succumbed to intestinal occlusion caused by fecaloma 4 weeks postoperatively, underwent immediate autopsy. Both S&S devices in this animal were firmly in place without dislodgement or visceral adhesions, and they were processed per protocol. The deceased pig was included among the two animals scheduled for short-term sacrifice (1 month).

**Fig. 7:**
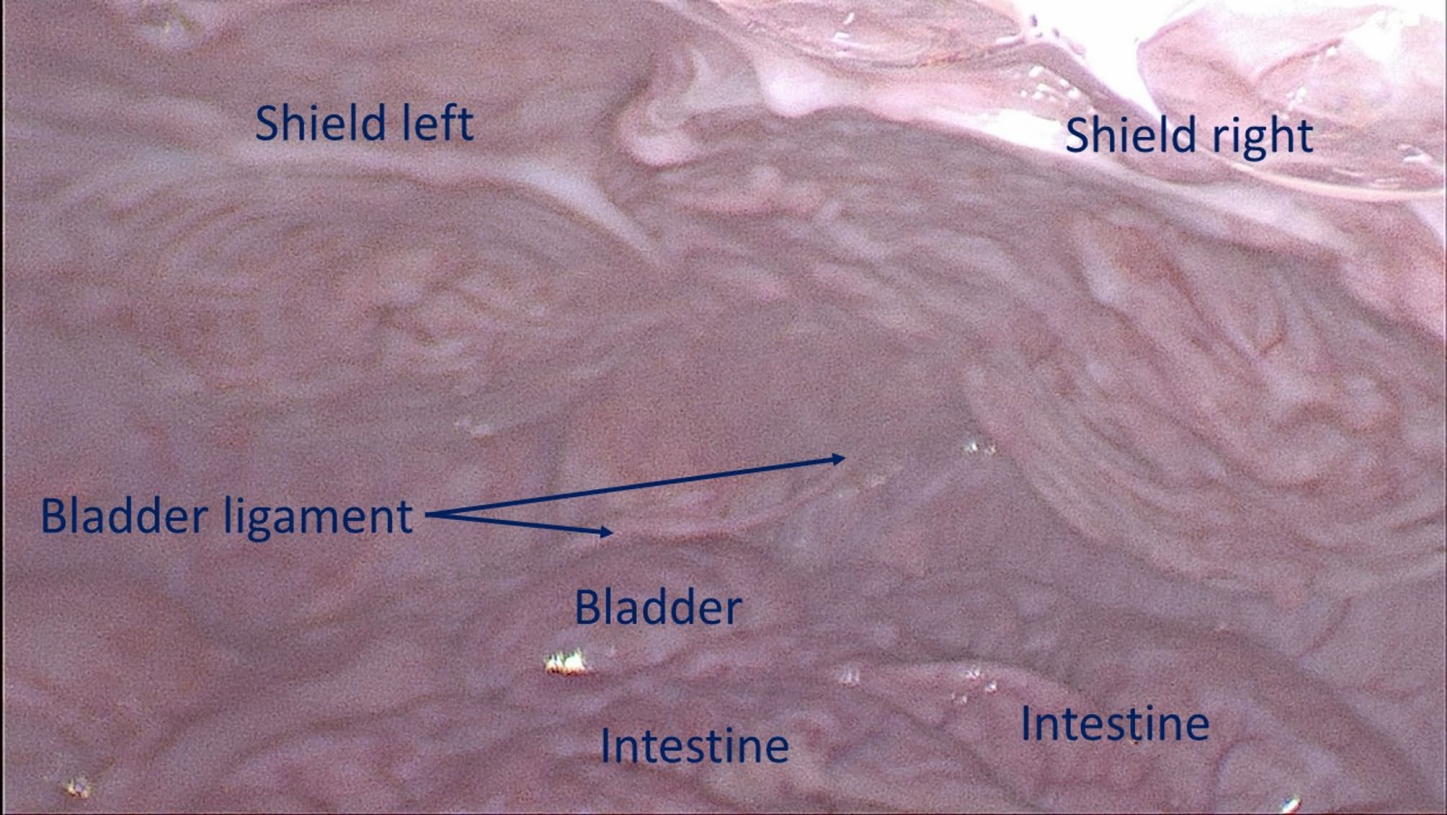
Intraabdominal view during laparoscopic control 6 months post-surgery: both shields placed over the defects are clearly covered by healthy peritoneum. No adhesion bridles to visceral organs are present. After the laparoscopic control the animal has been euthanized, the S&S device was then removed and sent to histological study.

**Fig. 8:**
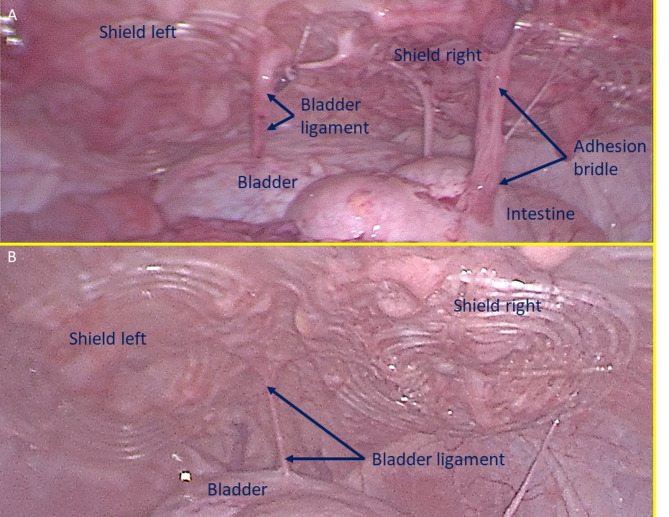
(**A**) Laparoscopic view of the shields positioned in the lower abdomen at the control 1 month postop. Exuberant mesothelial growth over both shields. The right sided shield shows an adhesion bridle to the intestine. (**B**) Laparoscopic control three months post-surgery. Both shields are increasingly covered by mesothelial tissue.

Ultrasound and laparoscopic controls during follow-up confirmed that all devices remained in their original positions without migration. Interaction between the shield and abdominal viscera showed no adhesions in any animals (Fig. 7), except for transient, thin adhesions observed in two animals during the one-month control (Fig. 8A). These adhesions resolved completely by the three-month follow-up (Fig. 8B). Substantially, at the time of sacrifice, no adhesions between the shield and abdominal viscera were observed in any animals.

### Macroscopic assessment at euthanasia

**Fig. 9:**
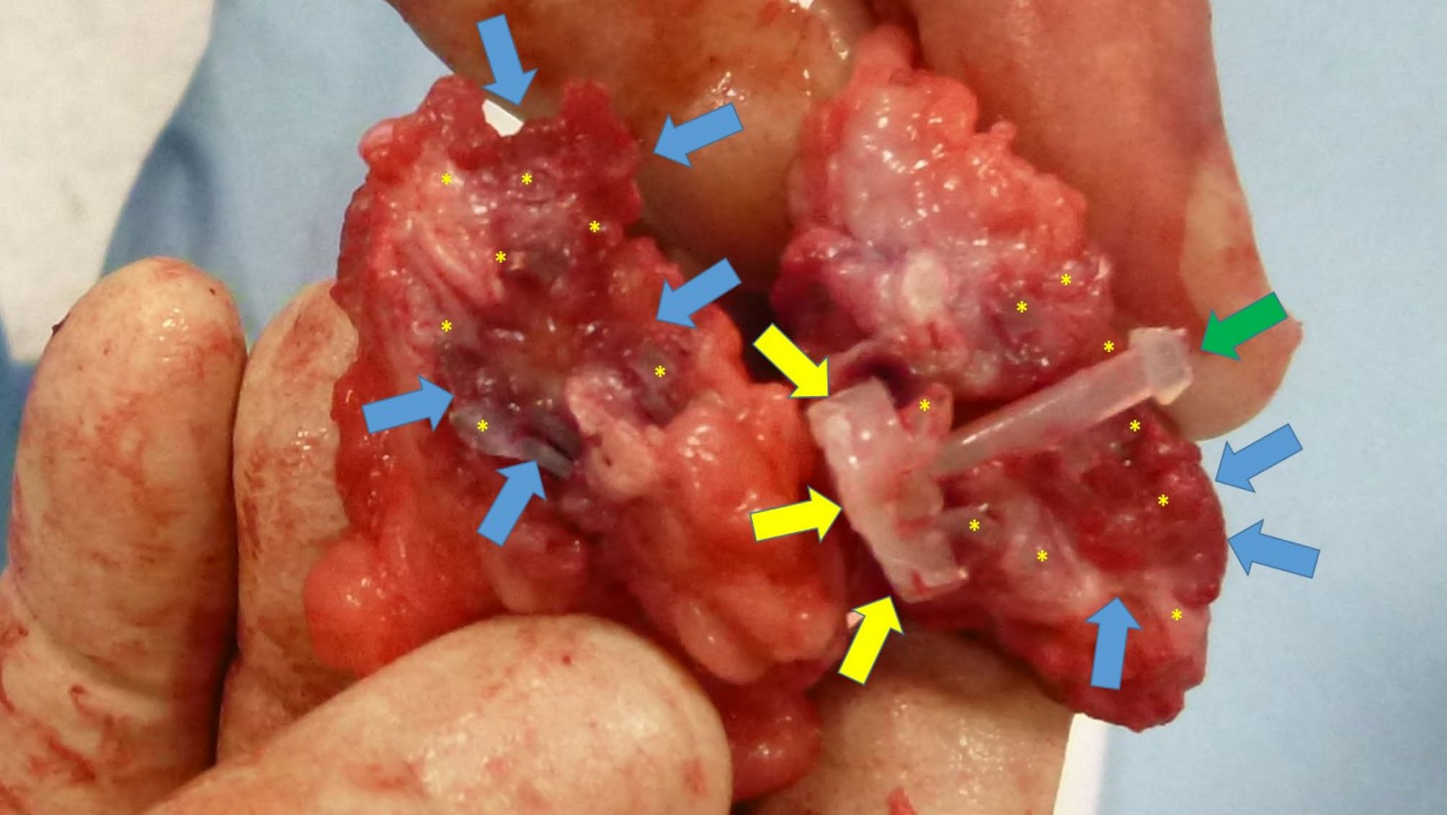
Scaffold of the S&S device explanted 6 months post-surgery and cut in half: the 3D chamber of the scaffold formed by the bent rays of the device (*) is evidently filled by newly ingrown fleshy tissue resembling viable muscular structure (blue arrows). Notable features include the distal portion of the mast with the conic stop (green arrow) and the button-like enlargement on the opposite side (yellow arrows).

Laparoscopic assessments prior to sacrifice showed that all shields were covered by healthy mesothelium (Fig. 7). Following explantation, the S&S devices were meticulously scarified to remove host native tissue from their boundaries to allow macroscopic evaluation. The excised scaffolds retrieved after 6 months or longer were found to be incorporated with newly developed fleshy tissue resembling muscular tissue (Fig. 9).

It should be noted that in Fig. 9 the device appears separable from the host abdominal wall because the surrounding tissue was intentionally removed during specimen preparation in order to focus histological evaluation on the newly ingrown tissue within the 3D scaffold. In vivo, both the stent and shield components were firmly integrated with the host tissue, as further confirmed by macroscopic inspection at euthanasia. All devices were then sent for histological examination.

### Histological evidences

#### Inflammatory response

**Fig. 10:**
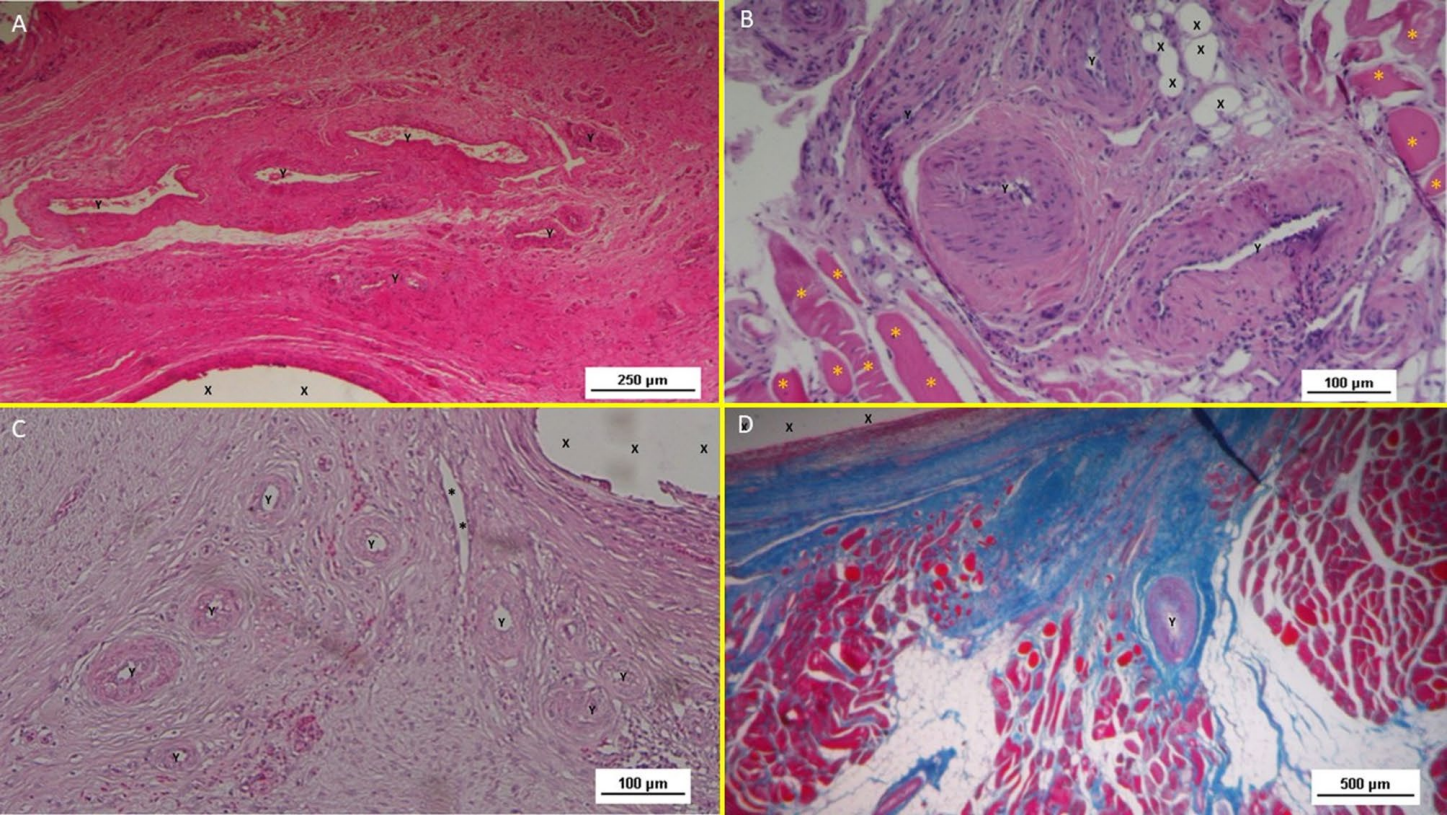
(**A**) Biopsy specimen excised 1 month post implantation: mild inflammatory infiltrate surrounding the structure of the S&S device (X). Of note the large vascular elements close to the S&S device (Y). HE 50X. (**B**) Biopsy specimen excised 3 months post implantation: absent inflammatory infiltrate surrounding the structure of the S&S device (X) and close to large convoluted arterial elements (Y) surrounded by a discrete amount of muscular fibers (*) HE 100X. (**C**) Biopsy from S&S scaffold removed 6 months postop: several mature arterial structures (Y) and one vein (*) in a context of lax and woven connective, close to the fabric of the S&S device. HE 100X.-10 (**D**) Biopsy sample excised 8 months post implantation. Mature muscle bundles (colored in red) developed close to the structure of the S&S device (X) surrounded by adipocytes (colored in white). A midsized arteriolar element is visible between the newly ingrown musculature (Y) AM 25X.

Histological analysis with HE staining revealed minimal inflammatory reactions in the early postoperative stage, characterized by limited lymphocytic and histiocytic elements (Fig. 10A). No more inflammation towards the S&S fabric was detected in mid, long, or long term stages (Fig. 10B, C).

#### Neoangiogenesis

Vascular development began early, with newly formed vessels observed within the 3D scaffold (Fig. 10A). Immature arteries and veins were evident in the short term, progressing structurally in the midterm (Fig. 10B) and achieving full development in the long term (Fig. 10C).

#### Connective tissue and neomyogenesis

**Fig. 11:**
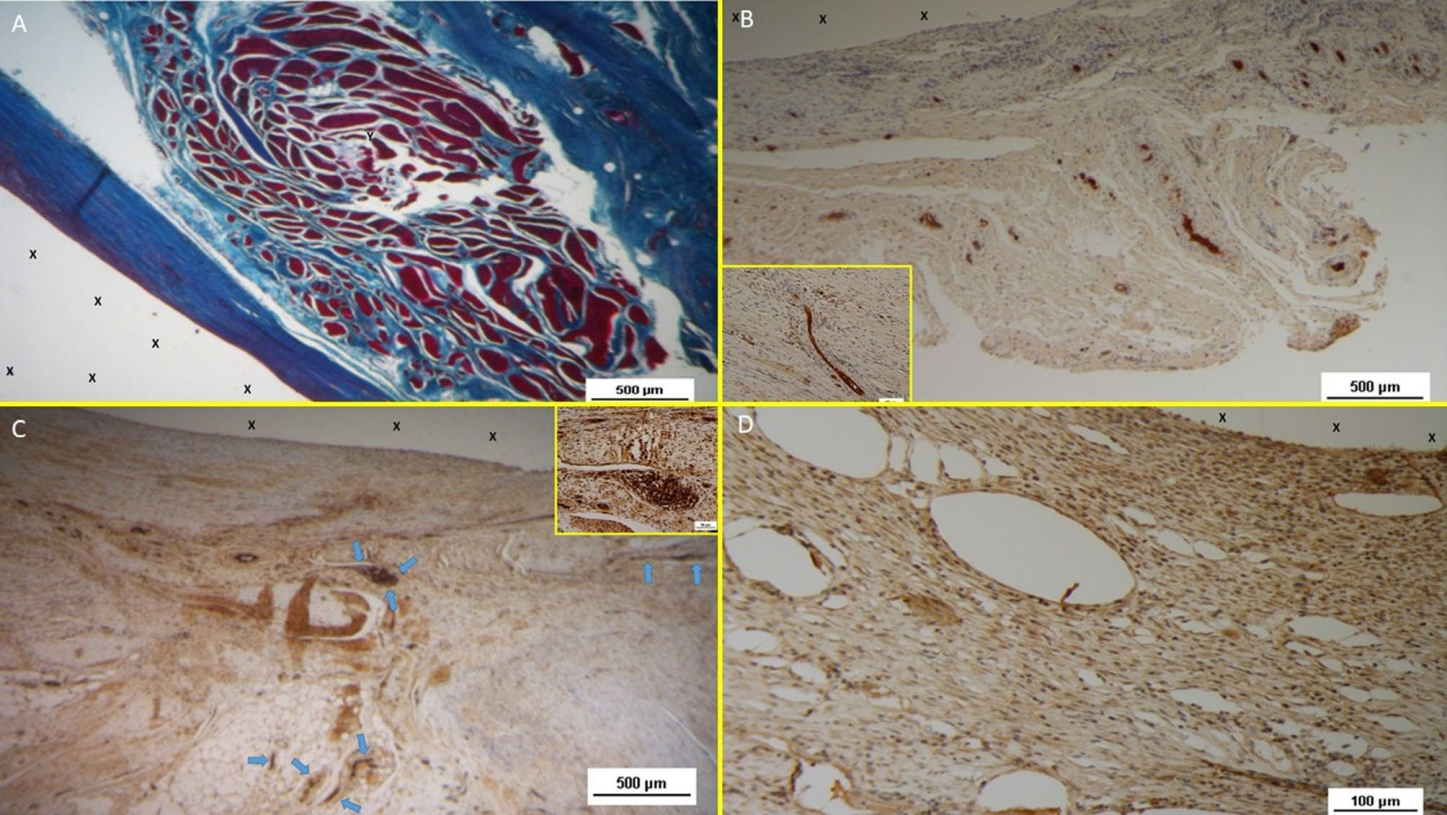
(**A**) Biopsy specimen removed eight months post implantation: well structured muscle bundles arranged in a fusiform element (colored in red) in a surround of healthy and compact connective (colored in blue) close to the fabric of the S&S device (X). AM 25X—(**B**) Biopsy sample removed from the 3D scaffold of the S&S device 3 months after implantation. The figure shows several nervous elements (colored in brown) close to the TPE fiber of the scaffold (X). NSE 25X—The microphotograph left below highlights in a nervous element higher magnification. NSE 200X—(**C**) Biopsy sample excised 6 months after implantation of the S&S device. The low magnification image shows several nervous elements (blue arrows) close to the TPE fiber of the scaffold (X). NSE 25x—The microphotograph right above details one nerve showing fully mature axon components and a healthy myelin sheath. NSE 200X—(**D**) Biopsy specimen excised 4 weeks after implantation showing several vascular structures (white elongated oval spots) in the stage of endothelial development close to the structure of the S&S device (X). VEFG 100X.

Azan Mallory trichrome staining demonstrated progressive incorporation of connective and muscular tissue. Initial development was observed early, with significant maturation by the midterm. In the long term, myocytes were fully developed within well-hydrated connective tissue while the muscle bundles exhibited complete structural maturity (Figs. 10D and 11A).

#### Neonervegenesis

Neo-neurogenic activity was detected early using NSE staining, revealing in the early stages the presence of immature nervous clusters. These clusters developed progressively in the midterm (Fig. 11B), achieving in the long-term full structural maturity with nervous bundles resembling the typical human nerve architecture (Fig. 11C).

### Immunohistochemical growth factor detection

**Fig. 12:**
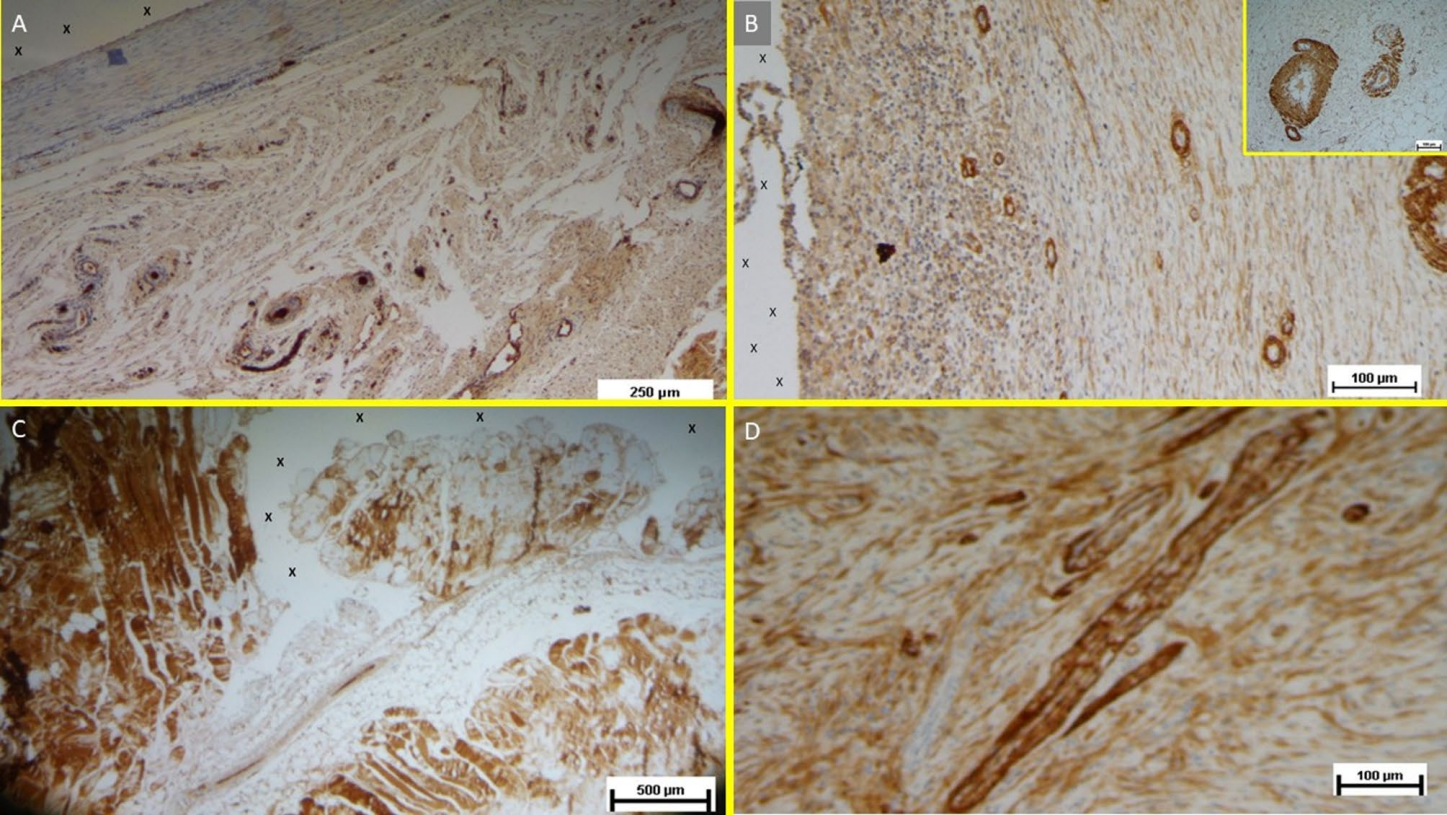
(**A**) Tissue sample removed from the 3D scaffold of the S&D device six months implantation. Numerous clusters of intense neo-angiogenesis (colored in brown) with well-developed vascular structures are clearly evident close to the device fabric (X). CD31 50X—(**B**) Tissue sample excised from the 3D scaffold of the S&S device 8 months post-surgery. Numerous venous elements showing well-constituted muscular layer are highlighted close to the scaffold fabric (X). SMA 100X—The high magnification microphotograph in the right upper corner depicts two arterial structures with a well-represented muscular layer (colored in brown). SMA 200X—(**C**) Biopsy sample excised from the scaffold of the S&S device 6 months post implantation. The image shows plenty of muscular elements (colored in brown) grouped in bundles close to the TPE structure of the device. NGF 25X—(**D**) Biopsy specimen excised from the 3D scaffold of the S&S device 6 months post-implantation. The image shows a mature elongated nervous structure (colored in brown) developed within the TPE scaffold. NGFRp75 100X.

- **Vascular Growth Factors**: Early VEGF-induced neovascularization (Fig. 11D) transitioned to a predominance of CD31-positive endothelial cells in the midterm, along with SMA-driven vascular maturation (Fig. 12A). Long-term specimens exhibited further SMA-mediated structural thickening, with complete vascular development (Fig. 12B).

- **Muscular Growth Factors**: NGF-positive elements indicated muscular development in the early stage, increasing in quantity and organization into bundles by the mid and long term (Fig. 12C).
- **Nervous Growth Factors**: NGFRp75-positive neural elements were initially sparse, correlating with immature nervous clusters. By the midterm, these elements increased, showing progressive myelin sheath development. Long-term specimens revealed mature nerve structures resembling human nerves (Fig. 12D).

## Discussion

Abdominal wall hernia repair primarily relies on flat meshes, which, while effective in reinforcing weakened abdominal wall, have notable limitations. These static meshes provoke a granulomatous foreign body response, leading to uncontrolled development of fibrotic scar tissue that hardens over time, potentially restricting muscle movement and causing discomfort. In cases where nerves are encapsulated by the exuberant ingrown of scar tissue, chronic pain may occur^19,20^.

Flat meshes often leave the hernia defect patent and usually require fixation to prevent dislodgement. Fixation is unphysiological since hinders the muscular movements and introduces risks such as tissue tearing, bleeding, hematoma formation, or mesh detachment, significantly increasing recurrence risks if dislodgment occurs^21,22^. Additionally, the extensive and time consuming tissue dissection required during the surgical procedure—whether via open or laparoscopic techniques—heightens the risk of iatrogenic injury, postoperative tissue swelling, prolonged pain, and delayed recovery.

Importantly, the foreign body response triggered by flat meshes, while promoting fibrotic scar tissue incorporation, fails to address the degenerative roots of hernia disease^10–14^. These roots, particularly in inguinal hernias, are often linked to chronic compressive damage that weakens the abdominal wall musculature. Conventional meshes reinforce but do not regenerate, leaving the abdominal wall barrier structurally deficient and prone to recurrence.

Abdominal wall hernia is nothing more than a hole in the muscle barrier. Starting from this consideration and reflecting pragmatically, the Stenting & Shielding (S&S) Hernia System was developed to address the typical challenges of conventional hernia repair by streamlining the procedure and promoting better outcomes. Its fixation-free design eliminates the need for sutures or tacks, reducing operative complexity and associated complications. Key features of the S&S device include:

1. Minimally invasive laparoscopic delivery without tissue dissection.
2. Permanent obliteration of the hernia defect to prevent recurrence.
3. An intraabdominal shield protecting neighboring areas from future herniation.
4. Dynamic compliance with abdominal wall movements, preventing visceral adhesions and fostering regeneration.

Inspired by vascular stents^23,24^, the device transforms from a cylindrical assembly into a 3D scaffold that fills the defect and is secured by an oval shield. The medical-grade polypropylene-based thermoplastic elastomer (TPE) ensures elasticity, durability, and adaptability to muscle movements without breaking. As documented in Table 1, the TPE material used for the experiment exhibits a tensile strength of 16 MPa and a strain at break of 650%, confirming its ability to undergo extensive reversible deformation while maintaining structural integrity^25,26^. Additional ISO-characterized properties, including tear strength (46 kN/m) and Shore A hardness (89), further support the material’s robustness and compliance^27–29^. These certified mechanical features provide objective evidence that the scaffold material can mimic the dynamic behavior of the abdominal wall, thereby supporting physiological motion while preventing device stiffening or shrinkage. Unlike conventional flat meshes, the S&S scaffold induces a probiotic biological response, leveraging its dynamic properties to foster tissue regeneration, including the formation of new muscle fibers, vascular elements, and nerves. This dynamic regeneration, as observed in other dynamic responsive scaffolds for hernia repair, seems to actively restore the integrity of the abdominal wall barrier while addressing the degenerative mechanisms underlying hernia disease^30,31^.

Adhesion formation, a common complication with static meshes, can lead to bowel obstruction and other issues. Flat meshes placed intraperitoneally are particularly prone to adhesions due to their passive nature^32,33^.

To address this, the S&S device employs TPE, a biocompatible material that transmits dynamic forces from abdominal wall movements to the shield, preventing the development of adhesion bridles. Preliminary experimental testing identified TPE as the optimal material due to its unique combination of elasticity and anti-adhesive properties, outperforming other tested substances, including polypropylene, polyester, and polyurethane.

The study demonstrated the secure retention of all 20 implanted S&S devices, with no migration or dislocation observed. At this regard is important to point out that, although the expansion of the stent within the hernia channel provides additional stability, the primary retention of the S&S Hernia System is ensured by the mechanical interaction between the conic stop(s) of the mast and the shield, which locks securely in place and prevents migration or dislodgement.

Regarding the macroscopic appearance of the intraabdominally placed shield, it is important to note that no adhesions with visceral organs were observed at the time of animal euthanasia, underscoring the biocompatibility of TPE and the role of dynamic compliance in adhesion prevention.

Macroscopic evaluations at animal sacrifice and after device excision, revealed the scaffold filled with newly formed tissue (Fig. 9), contrasting with the fibrotic response typical of conventional meshes. Histological analyses confirmed minimal inflammation, limited to the early period, and robust regeneration, with connective tissue, muscle fibers, nerves, and blood vessels progressively developing into the scaffold. Immunohistochemistry further highlighted growth factors (e.g., VEGF, CD31, SMA, NGF, NGFRp75) driving vascularization, myogenesis, and neural regeneration, demonstrating a probiotic biological response not observed in static mesh repairs^34,35^. This final feature provides additional evidence that the S&S device can effectively promote regeneration and re-establish the integrity of the muscular barrier. This property aligns perfectly with the goals of a device designed to address a degenerative condition such as an abdominal wall hernia.

Although the present experimental study was conducted with a single device size (4.5 cm), the system is intended to be industrially developed in a range of stent diameters (2.5–4.5 cm) to allow surgeons to select the most appropriate size according to the defect, with larger dimensions planned for future applications in very wide defects. Furthermore, the applicability of the S&S device is planned to be extended to a wide spectrum of hernia types—including inguinal, ventral, femoral, Spigelian, obturator, and incisional defects—once clinical use in humans begins.

## Conclusions

This study highlights the potential of the Stenting & Shielding (S&S) Hernia System in providing efficient, minimally invasive hernia repair. However, some limitations must be acknowledged. First, the sample size of 10 pigs with 20 implanted devices limits statistical power and generalizability. While this aligns with similar preclinical safety and efficacy studies^17^, resource constraints —particularly the costs associated with long-term animal care— required careful budget management.

Second, the study did not include direct comparisons with conventional hernia repair techniques, such as tension-free hernioplasty or laparoscopic mesh repair. Future randomized controlled trials in humans are essential to evaluate the S&S system’s comparative advantages and broader clinical applicability.

A further limitation of the present preclinical study is related to the anatomical orientation of the abdominal wall: while in pigs the wall is predominantly horizontal, in humans it is vertical. Although intra-abdominal pressure provides a stabilizing effect and no migration or displacement was observed in our series, we acknowledge that gravitational forces in the upright human abdomen may influence long-term device behavior. This aspect, already addressed with the first-generation ProFlor device in humans, warrants further confirmation in ongoing and future clinical studies with the S&S Hernia System.

Finally, this report focuses on the surgical and basic regenerative aspects of the device. A more comprehensive histological and immunohistochemical analysis will be detailed in subsequent publications to provide deeper insights into the biological mechanisms underlying the device’s performance.

Despite these limitations, the S&S Hernia System likely demonstrated notable efficacy and safety in this preclinical study. The device’s innovative design and material properties facilitated a fixation-free, dynamically responsive solution for defect obliteration, eliminating the risk of visceral adhesions and promoting tissue regeneration. The minimally invasive approach, requiring no surgical dissection, resulted in an intuitive, swift and easily reproducible procedure.

These findings suggest that the S&S system could manage various hernia types—including inguinal, femoral, Spigelian, obturator, and incisional hernias—without the surgical trauma associated with conventional techniques. By inducing true regeneration of connective tissue, muscles, vessels, and nerves, the S&S scaffold addresses the physiological, structural and biological deficiencies of conventional hernia repair methods. This regenerative capability ensures not only defect closure but also restoration of the abdominal wall’s functional integrity. If validated in human trials, this system could redefine hernia repair by offering a simplified repair concept rooted in pathophysiology and regeneration rather than reinforcement alone.

## Data Availability

No datasets were generated or analysed during the current study.

## Declarations

### Human and animal right

Animal experiments have been conducted in compliance with the guidelines of the Animal Care Protocol for Experimental Surgery issued by the Italian Ministry of Health.

## References

[1] Amato, G. State of the Art and future perspectives in inguinal hernia repair. In: Inguinal Hernia: Pathophysiology and Genesis of the Disease. Cham: Springer; 143–163. 10.1007/978-3-030-95224-2_8. (2022).

[2] Kingsnorth, A. & LeBlanc, K. Hernias: inguinal and incisional. *Lancet* **362**, 1561–1571 (2003). 14615114 10.1016/S0140-6736(03)14746-0

[3] LeBlanc, K. A. Complications associated with the plug-and-patch method of inguinal herniorrhaphy. *Hernia* **5** (3), 135–138 (2001). 11759798 10.1007/s100290100027

[4] Klosterhalfen, B., Klinge, U. & Schumpelick, V. Functional and morphological evaluation of different polypropylene-mesh modifications for abdominal wall repair. *Biomaterials* **19** (24), 2235–2246 (1998). 9884036 10.1016/s0142-9612(98)00115-x

[5] Klinge, U., Klosterhalfen, B., Muller, M. & Schumpelick, V. Foreign body reaction to meshes used for the repair of abdominal wall hernias. *Eur. J. Surg.* **65**, 665–673 (1999). 10.1080/11024159950189726 10452261

[6] O’Dwyer, P. J. et al. Randomized clinical trial assessing the impact of a lightweight or heavyweight mesh on chronic pain after inguinal hernia repair. *Br. J. Surg.* **92** (2), 166–170 (2005). 15584057 10.1002/bjs.4833

[7] Weyhe, D. et al. Experimental comparison of monofile light and heavy polypropylene meshes: less weight does not mean less biological response. *World J. Surg.* **30** (8), 1586–1591 (2006). 16855805 10.1007/s00268-005-0601-0

[8] Köckerling, F. et al. Open repair of primary versus recurrent male unilateral inguinal hernias: perioperative complications and 1-year follow-up. *World J. Surg.* **40** (4), 813–825 (2016). 26581369 10.1007/s00268-015-3325-9 PMC4767863

[9] Amato, G. et al. The septum inguinalis: a clue to hernia genesis? *J. Invest. Surg.* **31**, 1–9 (2018). 30380341 10.1080/08941939.2018.1497734

[10] Amato, G. et al. Histological findings of the internal inguinal ring in patients with indirect inguinal hernia. *Hernia* **13**, 259–262 (2009). 19234660 10.1007/s10029-009-0483-4

[11] Amato, G. et al. Nerve degeneration in inguinal hernia specimens. *Hernia* **15**, 53–58 (2011). 20953888 10.1007/s10029-010-0735-3

[12] Amato, G. et al. Damage to the vascular structures in inguinal hernia specimens. *Hernia* **16** (1), 63–67 (2012). 21739233 10.1007/s10029-011-0847-4

[13] Amato, G. et al. Muscle degeneration in inguinal hernia specimens. *Hernia* **16** (3), 327–331 (2012). 22015811 10.1007/s10029-011-0890-1

[14] Amato, G. et al. Histological findings in direct inguinal hernia. *Hernia* **17** (6), 757–763 (2013). 23288217 10.1007/s10029-012-1032-0

[15] Amato, G. et al. A new prosthetic implant for inguinal hernia repair: its features in a Porcine experimental model. *Artif. Organs*. **35** (8), E181–E190 (2011). 21752035 10.1111/j.1525-1594.2011.01272.x

[16] Amato, G. et al. Fixation free laparoscopic obliteration of inguinal hernia defects with the 3D dynamic responsive scaffold proflor. *Sci. Rep.* **12** (1), 18971 (2022). 36347998 10.1038/s41598-022-23128-6 PMC9643531

[17] Romano, G. et al. The dysloh study: comparative evaluation of the results between the proflor and Lichtenstein techniques for open inguinal hernia Repair-A randomized controlled trial. *J. Clin. Med.* **13** (18), 5530 (2024). 39337017 10.3390/jcm13185530 PMC11432422

[18] Kilkenny, C., Browne, W. J., Cuthill, I. C., Emerson, M. & Altman, D. G. Improving bioscience research reporting: the ARRIVE guidelines for reporting animal research. *PLoS Biol.* **8** (6), e1000412. 10.1371/journal.pbio.1000412 (2010). 20613859 10.1371/journal.pbio.1000412 PMC2893951

[19] Bay-Nielsen, M., Nilsson, E., Nordin, P. & Kehlet, H. Swedish hernia data base the Danish hernia data base chronic pain after open mesh and sutured repair of indirect inguinal hernia in young males. *Br. J. Surg.* **91**, 1372–1376 (2004). 15376186 10.1002/bjs.4502

[20] Mayer, F. et al. When is mesh fixation in TAPP repair of primary inguinal hernia necessary? A register-based analysis of 11,230 cases. *Surg. Endosc*. **30** (10), 4363–4371 (2016). 26886454 10.1007/s00464-016-4754-8 PMC5009149

[21] Watson, J. T., Webb, D. W., Stoikes, N. F. N. & Voeller, G. R. Affiliations expand fibrin sealant: A review of the History, Biomechanics, and current applications for prosthetic fixation in hernia repair. *Surg. Technol. Int.* **27**, 140–145 (2015). 26696538

[22] Jeans, S., Williams, G. L. & Stephenson, B. M. Migration after open mesh plug inguinal hernioplasty: a review of the literature. *Am. Surg.* **73** (3), 207–209 (2007). 17375772

[23] Sahu, R. A., Nashine, A., Mudey, A., Sahu, S. A. & Prasad, R. Cardiovascular stents: types and future landscape. *Cureus* **15** (8), e43438 (2023). 37711918 10.7759/cureus.43438 PMC10499059

[24] Borhani, S., Hassanajili, S., Tafti, H. A. & Rabbani, S. Cardiovascular stents: overview, evolution, and next generation. *Prog Biomater.* **7**, 175–205 (2018). 30203125 10.1007/s40204-018-0097-y PMC6173682

[25] ISO 37. ; Rubber, vulcanized or thermoplastic — Determination of tensile stress-strain properties. International Organiza-tion for Standardization, Geneva, Switzerland, 2017 (2017).

[26] ISO 815-1. ; Rubber, vulcanized or thermoplastic — Determination of compression set — Part 1: At ambient or elevated temperatures. International Organization for Standardization, Geneva, Switzerland, 2019. (2019).

[27] ISO 34 – 1. ; Rubber, vulcanized or thermoplastic — Determination of tear strength — Part 1: Trouser, angle and crescent test pieces. International Organization for Standardization, Geneva, Switzerland, 2015. (2015).

[28] ISO 7619-1. ; Rubber, vulcanized or thermoplastic — Determination of indentation hardness — Part 1: Durometer method (Shore hardness). International Organization for Standardization, Geneva, Switzerland, 2010. (2010).

[29] ISO 1183-1. :*2019; Plastics — Methods for Determining the Density of non-cellular plastics — Part 1: Immersion Method, Liquid Pyknometer Method and Titration Method* (International Organization for Standardization, 2019).

[30] Amato, G. et al. Physiologic cyclical load on inguinal hernia scaffold proflor turns biological response into tissue regeneration. *Biology* **12**, 434 (2023). 36979126 10.3390/biology12030434 PMC10045722

[31] Amato, G. et al. A regenerative scaffold for inguinal hernia repair: MR imaging and histological cross-evidence. *Int. J. Surg.* **96**, 106170 (2021). 34775110 10.1016/j.ijsu.2021.106170

[32] Matthews, B. D. et al. Assessment of adhesion formation to intra-abdominal polypropylene mesh and polytetrafluoroethylene mesh. *J. Surg. Res.* **114** (2), 126–132 (2003). 14559437 10.1016/s0022-4804(03)00158-6

[33] Turza, K. C. & Butler, C. E. Adhesions and meshes: synthetic versus bioprosthetic. *Plast. Reconstr. Surg.* **130** (5 Suppl 2), 206S–13S (2012). 23096974 10.1097/PRS.0b013e3182638d48

[34] Amato, G. et al. Dynamic responsive inguinal scaffold activates myogenic growth factors finalizing the regeneration of the herniated groin. *J. Funct. Biomater.* **13** (4), 253 (2022). 36412894 10.3390/jfb13040253 PMC9680268

[35] Amato, G. et al. Enhanced angiogenesis in the 3D dynamic responsive implant for inguinal hernia repair proflor. *Artif. Organs*. **45** (8), 933–942 (2021). 33529348 10.1111/aor.13926

